# Morbihan disease: treatment difficulties and diagnosis: a case report

**DOI:** 10.11604/pamj.2018.30.226.14440

**Published:** 2018-07-26

**Authors:** Alaa Aboutaam, Fouzia Hali, Kenza Baline, Meryem Regragui, Farida Marnissi, Soumiya Chiheb

**Affiliations:** 1 Department of Dermatology and Venereology, Ibn Rochd University Hospital, Casablanca, Morocco; 2 Department of Anatomy and Pathology, Ibn Rochd University Hospital, Casablanca, Morocco

**Keywords:** Morbihan disease, edema, face, treatment

## Abstract

Morbihan disease (MD) is a rare entity. Its nosography is unclear and its therapeutic management is difficult. We report a new case of MD. We report a case of a 51-year-old patient consulted in our department for a one year facial edema, erythema and papules reported by him, for which the patient was treated with cyclins, local and general corticotherapy, without improvement. The clinical examination found an important edema of the front and eyelids with an erythema of the cheeks covered with a few telangiectasias. The clinical, biological and histological findings lead to a diagnosis of Morbihan disease after excluding other diseases. Due to previous therapeutic failures, the patient was put on isotretinoin and furosemide with slight improvement. The particularity of our observation lies in the rarity and especially in the therapeutic difficulties encountered during this disease.

## Introduction

Morbihan disease is a rare entity. Its place in the nosography is uncertain. Sometimes called persistent solid facial edema, sometimes rosacea lymphoedematous. It may correspond to a particular clinico-pathological form of lymphoedema or rosacea [1, 2]. Its diagnosis is made in front of an erythematous edema persisting from the upper part of the face with a histology often nonspecific and this after the elimination of a clearly defined affections [1]. Its physiopathology remains poorly known thus making its treatment management difficult and not specific [3]. We report a case in which we highlight the diagnostic and therapeutic difficulties encountered during this condition.

## Patient and observation

**Figure 1:**
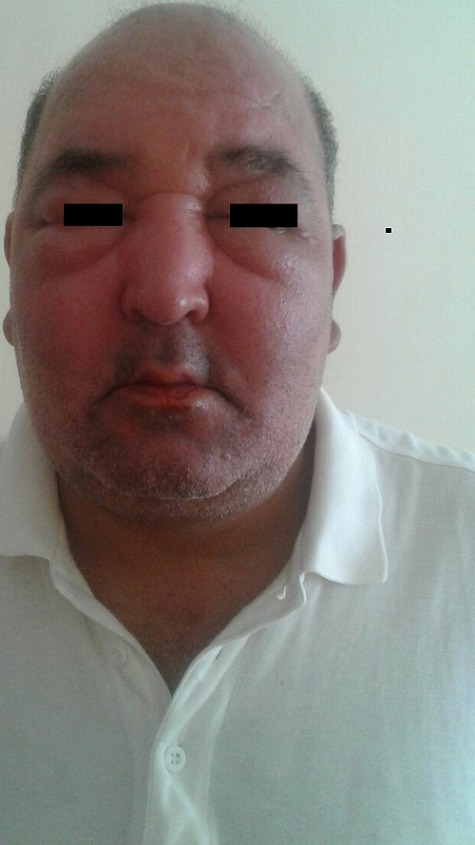
Edema of the face and eyelids with an erythematous cupboard cheeks

**Figure 2:**
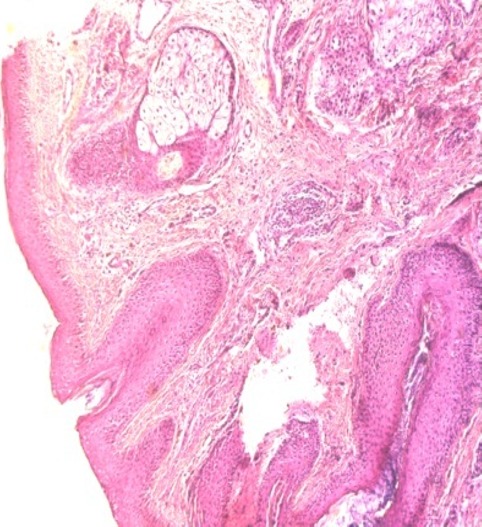
Perivascular dermatitis with vascular ectasia, a lymphocytic infiltrate

This is a 51-year-old patient with no specific pathological history who has consulted for edema of the upper side of the face predominant in the eyelids during the last year. The clinical examination showed a solid erythematous edema predominant over the forehead, eyelids and cheeks, which was solid in places and associated with telangiectasia without papular or pustular lesions (Figure 1). The patient reported papules in the begining of his disease. The histological study of the first cutaneous biopsy suggested lupus erythematosus. The immunological analysis (anti-nuclear antibody and native anti-DNA antibody) was negative. The dosage of muscle enzymes and the electromyogram showed no abnormalities. The diagnosis of lupus erythematosus was initially retained in view of the photographic distribution of the lesions and the histological data. A corticosteroid therapy (prednisone 1mg/kg/d) with progressively decreasing dose associated with chloroquine (200mg/d) was introduced but no significant improvement. The second cutaneous biopsy was therefore done, the histological study of which showed perivascular dermatitis with vascular ectasia, a lymphocytic infiltrate (Figure 2). Bacteriological and parasitological sampling for demodex were negative. Cerebro-facial magnetic resonance imaging (MRI) eliminated tumor compression or a loco-regional infectious cause. Skin test patches were also negative. The diagnosis of a Morbihan disease was ultimately retained in the clinical aspect, histology and elimination of other causes of facial edema (chronic lupus, dermatomyositis, chronic eczema and infectious causes). The patient was treated by isotretinoin (0.5 mg/kg/day) and furosemide 40 mg daily with slight improvement after 3 months.

## Discussion

The particularity of our observation lies in its rarity and especially in the diagnostic and therapeutic difficulties encountered during this condition. Morbihan syndrome was probably first described in 1956 by Schimpf [**1**]. And it was described in 1957 by French dermatologist Robert Degos in a native farmer of Morbihan (northwest region of France) as a condition characterized by erythematous chronic swelling of the forehead and eyelids, without Identifiable cause with non-specific histology [2]. Since this first case, few observations have been described. Its place in nosology is unclear. Is it a distinct condition or a clinico-pathological variety of rosacea or lymphedema or a rare complication of rosacea? Merklen, et al named this entity as massive persistent infiltration of the forehead with severe edema of the eyelids [4]. Or a clinical form of acne complication [5]. Indeed, its an association with the common form of rosacea that has been described in some observations [2, 3] as well as the presence of evocative histological images. This was also the case of our patient, since he had, in addition to facial edema, an erythema, telangeictasia and non present papules,which was reported by the patient at the begining of his disease all are evocative of a rosacea. The physiopathological mechanisms of Morbihan disease remain poorly understood. Different assumptions have been made. Some authors believe that the destruction of connective tissue perivascular dermis, especially elastin, resulting in loss of integrity of vascular walls with exudation of fluid causing swelling [1]. Others suggest lymphatic involvement [3]. The mast cells present in the perivascular infiltrate play a role in the development of edema. Indeed, this edema could be secondary to the permanent obstruction of the dermal lymphatics by the mast cells, or linked to the dermal fibrosis induced by these cells [1]. Nagasaki et al. Morbihan think the disease may result from a secondary lymphatic obstruction compression and lymph damage induced granulomas peri-lymphatic rosacea [3]. Another study demonstrated the presence of immunological nature of abnormalities in some patients, type of contact urticaria in association with local lymphatic disorders that could play a criticall role in the genesis of the Morbihan disease [6]. A recent study suggests that dilation of the superficial capillaries and venules and not of the lymphatic vessels, may be the cause of erythro-edema [7]. Clinically, the Morbihan disease is characterized by the gradual onset of systemic edema of solid consistency preferably sitting on the eyelids, forehead, glabella and cheeks [1].

The histology is often non-specific and does not allow us to retain the diagnosis. The observed images are characterized by a discrete hyperkeratosis with blanks of horny cones, epidermal atrophy, a discrete edema of middle and deep dermis, an inflammatory infiltrates of lymphocytes, histiocytes and neutrophils in perivascular and perifollicular, the capillary dilations Superficial fibrosis, perifollicular fibrosis and stromal edema [1]. Immunoglobulin deposits recalling a lupus band can be observed in direct immunofluorescence at the dermo-epidermal junction [1]. The diagnosis of Morbihan disease is retained before a set of clinical and paraclinical criteria and this after exclusion of a number of conditions. Indeed, several causes of chronic facial edema must be evoked and dismissed. Its etiologies are mainly represented by chronic lupus erythematosus, dermatomyositis, sarcoidosis, contact dermatitis, persistent solid facial edema complicating acne and Merkelson-Rosenthal syndrome in its atypical form, with involvement of the forehead and Eyelids [1]. Since the pathophysiology of Morbihan disease is poorly known, its management remains difficult and unspecific. Moreover, the literature shows the place of a panoply of treatments with contradictory results which are in their ineffective majorities, the case of our patient. These treatments include thalidomide [2], clofazimine, cyclines and corticosteroids alone or in combination with metronidazole [1, 7]. Isotretinoin used at high and long-term doses, alone or in combination with clofazimine or ketotifen, appears to be successful [1, 8]. However, isotretinoin may be ineffective in 15-20% of patients according to the literature [5]. In case of resistance to medical treatments, plastic surgery of reduction followed by lymphatic drainage, or blepharoplasty by laser CO2 in the forms with palpebral involvement are interesting [9]. Finally, Messikh et al Have recently reported the efficacy of diuretics alone or coupled with blepharoplasty. This efficacy has already been reported by a Turkish team using indapamide in combination with topical applications of 1% metronidazole [10].

## Conclusion

Several therapeutic variants have been proposed in the literature but they remain mostly nonsatisfactory. We illustrate through our observation the therapeutic difficulties encountered during this condition. Our patient received several medical treatments without any answer. A combinaton therapy of isotretinoin and furosemide gave a better reponse with transient regression of the edema than before with a patient comfort after 3 months treatment. Given the enormous psychological and socioprofessional impact of Morbihan disease, efforts must be made to better understand pathophysiological mechanisms in order to deduce more promising therapeutics in the future.

## Competing interests

The authors declare no competing interest.
